# Sodium alginate-bioglass-encapsulated hAECs restore ovarian function in premature ovarian failure by stimulating angiogenic factor secretion

**DOI:** 10.1186/s13287-021-02280-2

**Published:** 2021-04-01

**Authors:** Yating Huang, Zhijie Ma, Xiaojun Kuang, Qiuwan Zhang, Haiyan Li, Dongmei Lai

**Affiliations:** 1 The International Peace Maternity and Child Health Hospital, School of Medicine, Shanghai Jiao Tong University, 1961 Hua-Shan Road, Shanghai, 200030 People’s Republic of China; 2 School of Biomedical Engineering, Shanghai Jiao Tong University, 1954 Hua-Shan Road, Shanghai, 200030 People’s Republic of China; 3 Shanghai Key Laboratory of Embryo Original Diseases, 145 Guang-Yuan Road, Shanghai, 200030 People’s Republic of China; 4 Chemical and Environmental Engineering, School of Engineering, RMIT University, 124 La Trobe St, Melbourne, VIC 3000 Australia

**Keywords:** Premature ovarian failure, Human amniotic epithelial cells, Sodium alginate, Bioglass, Ovarian reserve, Chemotherapy

## Abstract

**Background:**

Human amniotic epithelial cells (hAECs) exhibit a strong capability to restore ovarian function in chemotherapy-induced premature ovarian failure (POF). However, the therapeutic efficacy of hAECs is usually affected by the limited number and proliferative ability of grafted hAECs in target organs. The transplantation of stem cells encapsulated in sodium alginate-bioglass (SA-BG) composite hydrogel has recently been shown to be an effective strategy for tissue regeneration. The current study aims to investigate the therapeutic potential of hAECs or hAEC-derived conditioned medium (CM) encapsulated in SA-BG in mice with chemotherapy-induced POF.

**Methods:**

C57BL/6 mice were intraperitoneally injected with chemotherapy drugs to induce POF. hAECs or CM were harvested and encapsulated in SA-BG composite hydrogel, which were transplanted onto the injured ovaries of mice with POF. Follicle development, granulosa cell function, and ovarian angiogenesis were evaluated by morphological methods. To further elucidate the effect of SA-BG-encapsulated hAECs/CM on vascularization, the tube formation of human umbilical vein epithelial cells (hUVECs) was conducted in vitro. Cytokine array and ELISA were used to analyze and quantify the effects of bioactive components released by SA-BG on the secretion of angiogenic factors by hAECs.

**Results:**

The transplantation of SA-BG-encapsulated hAECs/CM restored follicle development, repaired granulosa cell function, and enhanced ovarian angiogenesis in POF mice. The further study showed that SA-BG significantly promoted the tube formation of hUVECs in vitro. Moreover, encapsulating hAECs could facilitate the effect of SA-BG on inducing the formation of the capillary tube in a paracrine manner. In addition, we found that SA-BG extracts significantly enhanced the viability of hAECs and stimulated the secretion of pro-angiogenic factors of hAECs. Notably, compared with SA-BG/CM, SA-BG/hAECs achieve better therapeutic effects, possibly because stimulation of BG enhanced the viability and paracrine capacity of hAECs.

**Conclusions:**

The present study initially demonstrates that SA-BG-encapsulated hAECs or CM can exert a therapeutic effect on chemotherapy-induced POF mainly by protecting granulosa cell function and enhancing ovarian vascularization, which might provide a novel strategy for the delivery of hAECs for treating POF.

## Background

Premature ovarian failure (POF) is the cessation of ovarian function before the age of 40 years and accompanied by amenorrhea, sex steroid hormone deficiency, and elevated serum gonadotropin levels [1]. The incidence of POF is estimated to be as high as 1 in 100 women by the age of 40 years [2]. The etiology of POF is complex and includes genetic aberrations, autoimmune ovarian damage, iatrogenic factors, and exposure to toxic agents. A study reported that female cancer patients are more prone to experiencing ovarian insufficiency and infertility for prior exposure to chemotherapy drugs [3]. Thus, chemotherapy-induced POF is the most common form of POF in childbearing-age women who suffer malignancies [4].

Currently, it is still very difficult to restore damaged ovarian function with sequential hormone replacement therapy (HRT), which is commonly used to alleviate the symptoms of discomfort caused by hormone deficiency in POF patients [5]. Stem cells are self-renewing, have the capacity to differentiate into specific cell types under appropriate conditions, and exhibit strong paracrine potential, which brought new hope to the field of reproductive health [6]. Numerous studies have demonstrated that stem cells from different sources exhibit the potential to restore ovarian function in the chemotherapy-induced POF model [7].

hAECs derived from the amnion tissues of discarded placentas have attracted increasing attention in the field of regenerative medicine because they are easily isolated and their use is not affected by ethical debates [8]. Our previous studies have demonstrated the safety and efficacy of the use of hAECs in the chemotherapy-induced POF mouse model and hAECs could restore ovarian function and fertility [9]. In addition to the homing and differentiation of grafted hAECs in injured ovaries [10], paracrine cytokines exert substantial effects in repairing ovarian function by inhibiting granulosa cell apoptosis [11] and promoting angiogenesis [12]. We also have elucidated that hAEC-derived exosomes inhibit granulosa cell apoptosis by transferring functional microRNAs, thus contributing to the recovery of ovarian function [13].

In most animal studies, the routes of stem cell transplantation usually include tail vein [14], intraperitoneal [15], and ovarian orthotopic injections [11]. Although tail vein injection is the most widely used transplantation method for delivering cells to the recipient, most engrafted cells are trapped in the lungs and do not reach the target organs [16], which might cause pulmonary embolism issues and limit the cell dose for application. The intraperitoneal injection of stem cells is associated with the problems of low efficiency in target organs and rapid loss [17]. On the other hand, the ovaries of mice with POF usually shrink and exhibit severe fibrosis, which hinders the homing of grafted stem cells delivered via orthotropic injection. Additionally, the tissue microenvironment in injured ovarian is not suitable for the survival of grafted stem cells and possibly interrupts cytokine production by these stem cells. Thus, the selection of the optimum modality for transplantation is vital for the homing and survival of stem cells and affects stem cell-mediated functional recovery. Therefore, it is of great significance to explore new techniques for stem cell transplantation in order to further enhance the potential of hAEC-based therapy in POF animal models.

With the development of regenerative medicine, the precise transport of stem cells to the injured sites can be achieved by biomaterials. Studies have found that the transplantation of umbilical cord-derived mesenchymal stem cells on a collagen scaffold improves ovarian function in the POF mouse model [18] and activates dormant follicles in ovaries of POF patients with a long history of infertility [19]. Sodium alginate (SA) is a natural gelling agent that has been demonstrated to have good biological properties for cell delivery by supporting cell adhesion and proliferation and prolonging the time for which stem cells remain in the application site [20, 21]. Bioglass (BG) has been extensively proven to have bioactivities for promoting osteogenesis and angiogenesis/vascularization [21–23]. In our previous studies, sodium alginate-bioglass (SA-BG) composite hydrogel enhances vascularization by upregulating angiogenic growth factor expression in endothelial cells and contributes to skin tissue regeneration [24, 25]. Moreover, SA-BG composite hydrogel containing bone marrow stem cells (BMSCs) enhances osteochondral regeneration mainly by promoting vascularization [26]. However, it is not clear whether SA-BG composite hydrogel could be used to carry hAECs to achieve the precise transport of therapy.

In the current study, we established a new approach for the transplantation of hAECs and hAEC-conditioned medium (CM) encapsulated in SA-BG, which can be used as coverage onto the damaged ovaries of mice with POF. Furthermore, ovarian function and the underlying molecular mechanisms were evaluated by series of in vitro and in vivo experiments. We aim to demonstrate whether the transplantation of hAECs or CM encapsulated in SA-BG could serve as a new strategy for restoring ovarian function in the POF mouse model.

## Materials and methods

### Ethics

After obtaining informed consent, human placental tissues were collected from healthy women who tested negative for HIV-1, hepatitis B, and hepatitis C. The protocol for isolating hAECs was approved by the Institutional Ethics Committee of the International Peace Maternity and Child Health Hospital. All the animal experimental procedures were approved by the Institutional Animal Care and Use Committee of Shanghai and were performed in accordance with the National Research Council Guide for the Care and Use of Laboratory Animals. Efforts were made to limit the number of animals used and minimize the suffering of animals in the study.

### Isolation and culture of hAECs and preparation of hAEC-CM

As previously described [10], the amnion was bluntly separated from the placental tissue, dissected into segments, and digested with 0.25% trypsin/EDTA (Biological Industries, Kibbutz Beit Haemek, Israel). The cells were centrifuged at 1000 rpm for 5 min, resuspended in phosphate-buffered saline (PBS), and filtered through a 40-μm filter (Millipore, Billerica, MA, USA). Then, the hAECs were seeded onto 100-mm plates containing DMEM/F12 with 10% fetal bovine serum (Gibico, Carlsbad, CA, USA) and 10 ng/ml recombinant human epidermal growth factor (ProSpec, Mississauga, Ontario, Canada). The incubator was set at 37 °C and contained 5% CO_2_.

The CM was collected from hAECs as previously described [12]. The hAECs were cultured in a 100-mm culture dish. When the cells reached 70–80% confluence, the complete medium was replaced with 10 ml of serum-free DMEM/F12. After 24 h, the supernatant was collected and passed through a 0.45-μm filter (Millipore) to remove the cell debris. Then, each 10-ml filtered supernatant was further concentrated to a final volume of 1 ml by centrifugation for 30–60 min at 3200*g* in a prerinsed centrifugal filter tube (3 kDa; Amicon Ultra-15; Millipore) and stored in − 80 °C freezer.

### Preparation of SA-BG, SA-BG/hAECs, SA-BG/CM composite hydrogel, and the extracts of SA-BG composite hydrogel

As described in a previous study [26], SA from brown algae (medium viscosity; Sigma, USA) was sterilized with ultraviolet light before being dissolved in deionized water or concentrated hAEC-CM to produce a 2% (w/v) SA solution; this solution was stored at 4 °C. Bioglass (BG) powders with a diameter of 5–20 μm were purchased from Kunshan Chinese, Technology New Materials Co., Ltd. (Kunshan, Jiangsu, China) and sterilized with ultraviolet light for further use. Gluconic acid δ-lactone (GDL, > 99.0%) was obtained from Sigma-Aldrich and sterilized with ultraviolet light for further use. Briefly, 1 ml of 2% (w/v) SA solution, 20 mg of BG powder, and 20 mg of GDL (Sigma) were manually mixed in an injector with a t-branch pipe to produce a homogeneous solution. Then, the mixed solution was injected into a Teflon mold with a cylindrical shape, and a SA-BG composite hydrogel was obtained after gelation.

In the animal experiments, a total of 6 × 10^7^ hAECs in 0.2 ml PBS were gently encapsulated in a volume of 2 ml 2% (w/v) SA solution, BG, and GDL gently as SA-BG/hAECs composite hydrogel (SA-BG/hAECs) for use. Forty milligrams of SA was dissolved with a volume of 2 ml concentrated hAEC-CM to produce a 2% (w/v) SA-CM solution before the day of transplantation surgery and stored at 4 °C for further use as SA-BG/CM composite hydrogel (SA-BG/CM). Ultimately, approximately 100 μl composite was topically administered onto each ovary.

To prepare extracts of the SA-BG composite hydrogels, a 1-cm^3^ sample of the SA-BG composite hydrogel described above was immersed in 10 ml DMEM/F12 medium (Gibico) at 37 °C for 24 h. After incubation, the solution was removed from the dish, centrifuged at 1000 rpm (Cence L530) to remove the hydrogel debris, and filtered with a 0.22-μm filter to sterilize the solution. The extracts of the SA-BG composite hydrogel were stored at 4 °C for further use.

### Identification and characterization of hAECs

Flow cytometry was used to identify the surface marker expression of the hAECs. After incubation with primary antibodies, including antibodies against HLADR (1:1000, BioLegend, San Diego, USA), CD146 (mesenchymal marker, 1:1000, BioLegend), CD34 (hematopoietic marker, 1:1000, BioLegend), CD324 (epithelial marker, 1:1000, BioLegend), and SSEA4 (pluripotent marker, 1:1000, BioLegend), the hAECs were washed and resuspended in staining buffer. Flow cytometry was immediately performed using an FC500 flow cytometer (BD Pharmingen, San Diego, CA, USA).

For further characterizing the hAECs, immunofluorescence staining was performed. Paraformaldehyde-fixed cells were blocked with 10% goat serum. Then, the cells were incubated with the following primary antibodies: cytokeratin 18 (CK18, an epithelial marker, 1:200, Boster Biological Technology, Wuhan, China) and TRA-1-60 (stem cell marker, 1:1000, Cell Signaling Technology, CST, Beverly, MA, USA). After washing, the hAECs were incubated with secondary antibodies conjugated to Alexa Fluor 488 and 594 (1:3000, CST) and counterstained with DAPI (Abcam, Cambridge, UK). Finally, the immunofluorescence signals were observed with a Leica DMI3000 microscope (Heidelberg, Germany). The negative control cells received identical treatments, except primary antibodies were omitted, and exhibited no specific staining.

### Establishment of premature ovarian failure (POF) mouse model

**Fig. 1:**
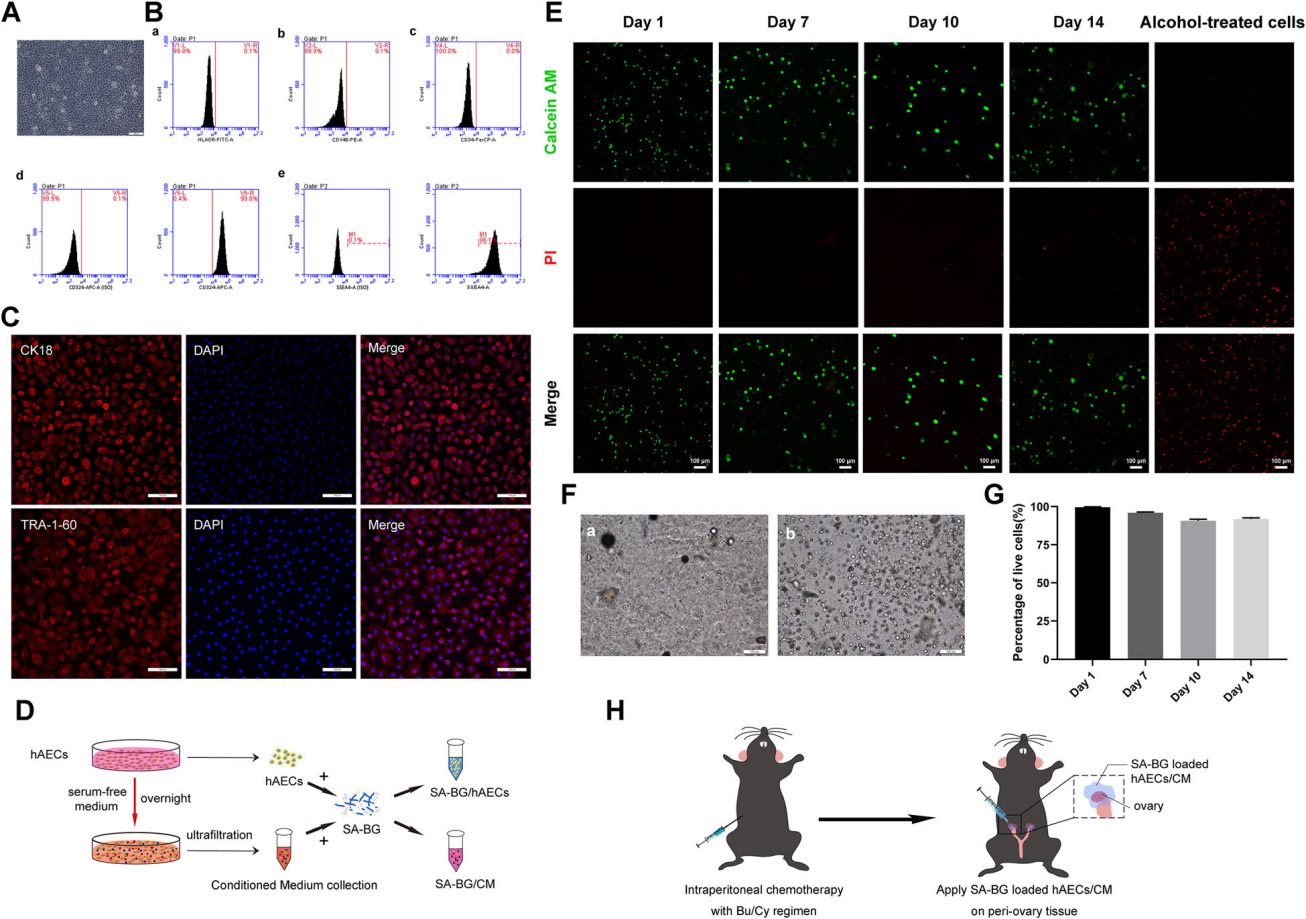
Characterization of hAECs, detection of the survival hAECs encapsulated in SA-BG, and schematic illustration of the surgical procedure. **a** The morphology of cultured hAECs was observed under a microscope. Scale bar 100 μm. **b** Flow cytometry analysis of cell surface markers on hAECs. The isotypes (ISO) of SSEA4 and CD324 were used as negative controls. **c** Immunostaining images showed the high expression of epithelial marker (CK18) and stem cell marker (TRA-1-60). Scale bar 100 μm. **d** The fabrication method of SA-BG-loaded hAECs and CM. **e** Representative live/dead images of hAECs encapsulated in SA-BG at days 1, 7, 10, and 14, respectively. hAECs encapsulated in SA-BG treated with 70% alcohol were positive for PI. Live cells were shown green color and dead cells were red color. Scale bar 100 μm. **f** Bright field image of hAECs capsulated in SA-BG at day 1 (**a**) and 14 (**b**). Scale bar 100 μm. **g** The percentage of live cells to total cells. **h** Schematic of the experimental procedure for the transplantation of SA-BG-loaded hAECs/CM into mice with chemotherapy-induced POF

Female C57BL/6 mice (8 weeks old, *n* = 60) were obtained from the Shanghai Experimental Animal Center of the Chinese Academy of Sciences. The POF model was established according to the method previously described [12]. Briefly, a total of 50 mice were intraperitoneally (*i.p.*) injected with chemotherapy drugs, including busulfan (Bu, 30 mg/kg, Sigma) and cyclophosphamide (Cy, 120 mg/kg, Sigma), to induce ovarian damage. The mice in the Sham group were injected with an equivalent volume of PBS (*n* = 10). One week after chemotherapy injection, transplantation surgery was performed. All the surgical operations were performed under anesthesia, and the ovaries in the different treatment groups (including the SA-BG group, SA-BG/hAECs group, and SA-BG/CM group, *n* = 10) were covered with the composites described above with needleless syringes through small abdominal incisions (Fig. 1h). The ovaries were gently returned when the hydrogels solidified. In the Bu/Cy control group, the animals underwent sham operation. Simple continuous sutures (Vicryl 4-0, Ethicon, USA) were used to close the abdomens, and the skin was sutured intermittently.

### Histology and follicle counting

The mice were sacrificed at day 3 (*n* = 30) and day 28 (*n* = 30) after transplantation. All the left ovaries were collected, fixed, dehydrated, embedded in paraffin, and serially sectioned at a thickness of 5 μm. The sections were deparaffinized, hydrated, and washed. Hematoxylin and eosin (H&E) staining was performed to observe the morphologic structures of the ovaries.

To count the number of follicles in the ovary, follicles were divided into four classes based on standard established methods [27]. Briefly, blind follicle counts were conducted by two independent researchers in every fifth section of the ovary. Primordial follicles were defined by granulosa cells surrounding a single fusiform oocyte. Primary follicles were surrounded by at least three cubic-shape granulosa cells. Secondary follicles appeared to be surrounded by at least two layers of granulosa cells with no follicular cavity. Antral follicles contained at least two layers of granulosa cells and exhibited evidence of follicular cavity formation. In each section, only the follicles that contained oocytes with clearly visible nuclei were counted.

### Immunohistochemical (IHC) staining

After deparaffinization and hydration, the ovarian sections were placed in sodium citrate solution and boiled for antigen retrieval. The following process was performed using an IHC kit (Abcam). Briefly, sections were treated with hydrogen peroxide and blocked, followed by incubation with primary antibodies against von Willebrand factor (vWF, newly formed vessels marker, 1:500, Boster), CD34 (endothelial marker, 1:2000, Abcam), and DDX4 (oocyte marker, 1:2000, Abcam). Then, the sections were washed and sequentially incubated with biotinylated anti-mouse/rabbit IgG, streptavidin peroxidase, and DAB chromogen solution, and finally, counterstained with hematoxylin. The negative control samples received identical treatment, except primary antibodies were omitted, and exhibited no specific staining. For quantification, four ovarian sections from each mouse were subjected to IHC, and three randomly selected high-power fields in each section were analyzed. The final data represent the average microvessel density (MVD).

### Immunofluorescence staining

Immunofluorescence staining was used to observe the proliferation of granulosa cell in the injured ovaries. Following deparaffinization and hydration, sections were blocked with solution containing 0.1% Triton X-100 and 10% goat serum and then incubated with anti-Ki-67 antibody (1:1000, Abcam). After washing, the sections were incubated with anti-mouse IgG-fluorescein isothiocyanate (FITC, 1:1000, CST) secondary antibody. The nuclei were stained with DAPI. After washing, the sections were covered with coverslips. The fluorescence signals were observed with confocal laser scanning microscopy (Leica).

### TUNEL staining

In situ Cell Death Detection Kit (R&D, Minneapolis, MN, USA) was used to detect apoptosis in the ovarian sections. Briefly, the dewaxed and hydrated sections were treated with proteinase K working solution for 20 min. All the sections were incubated with 50 μl TUNEL reaction mixture (45 μl dUTP labeled with fluorescein and 5 μl TdT). After washing, the sections were counterstained with DAPI and mounted with glass cover slides for observation with a confocal microscope (Leica).

### Calcein-AM/PI staining

Calcein-AM/PI staining kit (Yeasen, Shanghai, China) was used to observe the live/dead cells in SA-BG composite hydrogel. A total of 6 × 10^7^ hAECs in 0.2 ml PBS were gently mixed with 2 ml SA-BG and cultured in a complete medium for observation. The medium was changed every 2 days. Calcein-AM/PI staining was conducted at days 1, 7, 10, and 14. The cells were incubated with Calcein-AM (2 mM) and PI (1.5 mM), and then, the green/red fluorescence signals were observed under a confocal microscope (Leica). Six randomized images (× 100) were captured, and positive fluorescence signals were counted. The mean value was obtained to represent the cell viability at each time point.

### Tube formation assay

Human umbilical vein endothelial cells (hUVECs) were seeded in the bottom chamber in poly-L-lysine-coated 24-well plates at a density of 3 × 10^4^ cells per well. According to the experimental design, SA-BG, hAECs, SA-BG/hAECs, and SA-BG/CM were placed in the upper inserts. Then, the hUVECs were cocultured with the different upper chambers. Following coculture for 3 days, images of the capillary-like network were captured with a light microscope (Leica DM 2500), and the numbers of tubes and nodes were manually calculated.

### Cell Counting Kit-8 (CCK-8) assay

Cell viability was evaluated by CCK-8 assay (Beyotime, Shanghai, China). Briefly, hAECs were seeded in 96-well plates (5 × 10^3^ cells/well) and cultured with SA-BG extracts. The hAECs in the control group were cultured normally, and the culture media were changed every 2 days. At the indicated time points (days 1, 3, 7, and 10), CCK-8 solution was added to each well, and hAECs were further incubated for 1 h at 37 °C. The absorbance was measured at 450 nm using a microplate reader (Thermo Labsystems, Waltham, MA, USA).

### Western blotting

Ovary samples were obtained from mice sacrificed at day 3 after surgery and were lysed using RIPA (Beyotime, Shanghai, China). About 10 μg of total protein lysates was separated by 12.5% SAS-PAGE gel and transferred onto polyvinylidene fluoride (PVDF, Millipore) membrane. Membranes were blocked with 5% skim milk and incubated in the following primary antibodies: rabbit monoclonal anti-PCNA (1:1000; CST), mouse monoclonal anti-beta-tubulin (1:2000; Yeasen), rabbit monoclonal anti-cleaved Caspase 3 (1:1000; CST), and rabbit monoclonal anti-Caspase 3 (1:1000; CST). Secondary goat anti-mouse horseradish peroxidase (HSP)-conjugated antibody was used (1:3000; Yeasen). Following, blots were detected using a chemiluminescence kit (Millipore) and measured with ImageJ software (version 1.50i, NIH, Bethesda, MD, USA).

### Quantitative real-time PCR (qRT-PCR)

Total RNA was extracted from the hAECs according to a standard protocol. Then, the purity and concentration of the RNA were detected with a NanoDrop 2000c (Thermo Scientific, Bonn, Germany), and equal amounts of RNA were reverse transcribed into cDNA according to the kit manual (Takara, Dalian, China). The PCR primers were designed according to the cDNA sequences in the NCBI database (Supplementary Table 1). The cycling conditions for the PCR machine were as follows: 95 °C for 5 min and 60 °C for 34 s for 35 cycles. The gene expression levels were evaluated using the delta-delta CT method and standardized to Actin expression levels.

### Cytokine array and ELISA

The conditioned medium harvested from the hAECs or SA-BG extract-treated hAECs was analyzed using Human Angiogenesis Array (R&D), according to the manufacturer’s instructions. Images were captured by Chemi Scope 6300 system, and semiquantitative analysis of the relative intensity of the spots was performed with the HLImage analysis program (R&D).

To quantify the angiogenic factors, human insulin-like growth factor binding proteins-2/3 (IGFBP-2/3), tissue inhibitor of human matrix metalloproteinases (TIMP-1), human thrombospondin protein-1 (TSP-1), human matrix metalloproteinase 9 (MMP-9), human coagulation factor III, and human vascular endothelial growth factor (VEGF) released from hAECs treated with or without SA-BG extracts into the CM were measured by ELISA kits (Lengton, China, Shanghai), according to the manufacturer’s protocol.

### Statistical analysis

The results were graphed and analyzed using the GraphPad Prism version 8.0. The data are expressed as the mean ± SEM. Statistical significance was determined by one-way analysis of variance (ANOVA) followed by Dunnett’s test. The differences were considered to be statistically significant at *p* < 0.05.

## Results

### The establishment of hAECs encapsulated with SA-BG composite hydrogel and transplantation in the POF mouse model

Cultured hAECs exhibited the cobble stone-like appearance of epithelial cells (Fig. 1a). Flow cytometry displayed that hAECs expressed SSEA4 and CD324, but did not express HLADR, CD146, and CD34 (Fig. 1b). Immunofluorescent assay further showed that both CK18 and TRA-1-60 strongly expressed in hAECs (Fig. 1c).

To determine whether the transplantation of hAECs or CM encapsulated in SA-BG could serve as a new strategy to restore ovarian function, we prepared the SA-BG composite hydrogel containing hAECs and CM as shown in Fig. 1d. Before transplantation, we firstly assayed the survival status of hAECs cultured in SA-BG hydrogel with live/dead cell staining. Results showed that most of hAECs in SA-BG were positive for calcein-AM (alive, green) and only a small number of hAECs were positive for PI staining (dead, red) (Fig. 1e). Furthermore, the morphological results displayed that the hAECs in SA-BG exhibited a round shape (Fig. 1f). Statistical results showed that the percentage of live cells (calcein-AM-positive cells) was 99.604 ± 0.279 (%) at day 1 and 91.848 ± 0.750 (%) at day 14 (Fig. 1g). These results demonstrated that hAECs could survive in SA-BG composite hydrogel for at least 14 days.

Then, we established a chemotherapy-induced POF mouse model to investigate whether SA-BG-encapsulated hAECs/CM have the potential to restore ovarian function. As shown in Fig. 1h, bilateral ovaries of the mouse were exposed and covered with SA-BG, SA-BG/hAECs, or SA-BG/CM composite hydrogel, respectively, at 7 days after chemotherapy treatment. No adverse reactions or death occurred in the mice after transplantation.

### SA-BG-loaded hAECs/CM transplantation restored follicle development in the POF mouse model

**Fig. 2:**
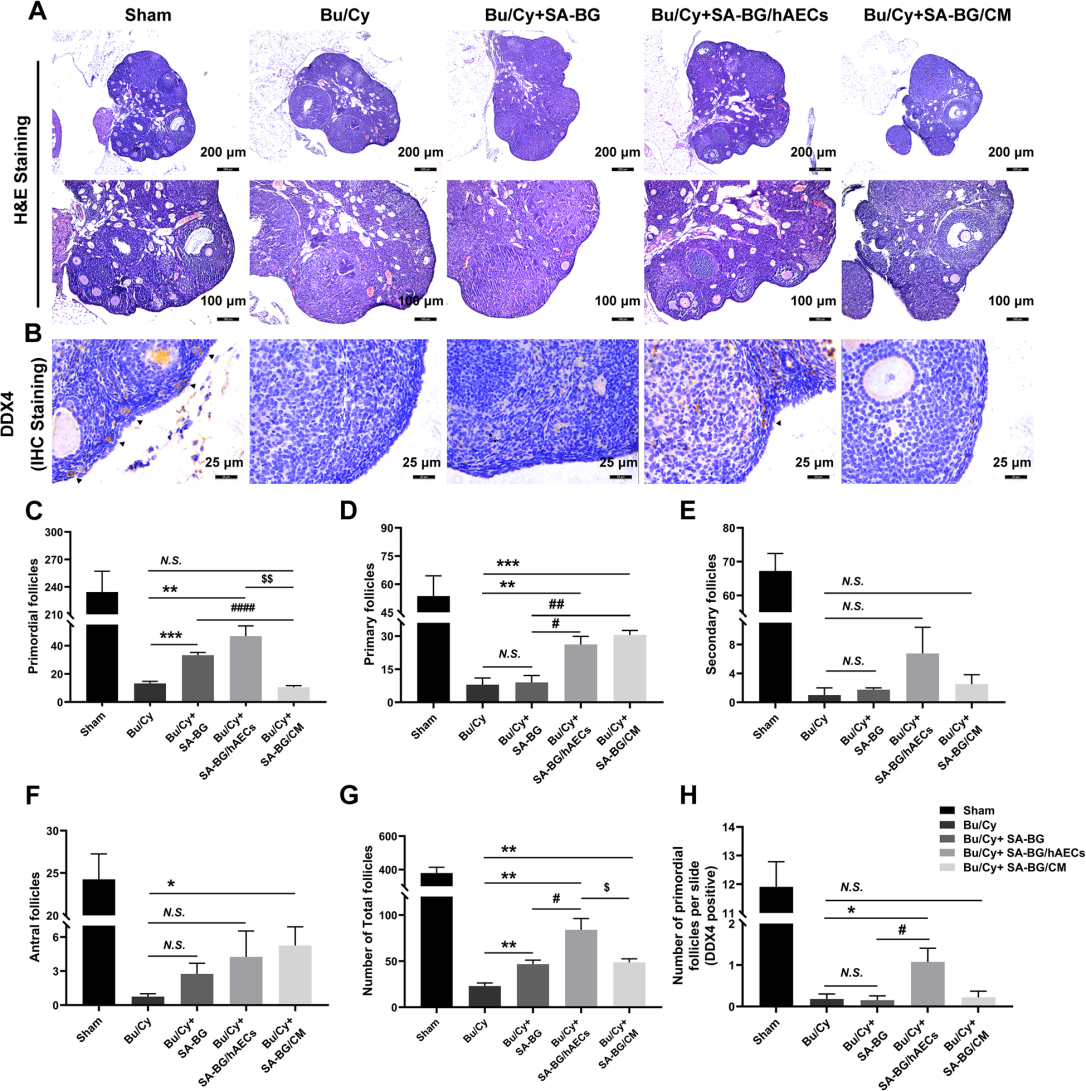
Morphological observation and follicle count in ovaries of mice at day 28 after transplantation. **a** Representative photographs of ovarian sections stained with H&E from different treatment groups. Scale bars 200 μm and 100 μm. **b** Immunochemical staining of DDX4 in ovarian sections from different groups. The bold black arrowhead indicated DDX4-positive primordial follicles. Scale bars 25 μm. **c**–**f** Column displayed the number of calculated primordial follicles, primary follicles, secondary follicles, and antral follicles in the whole ovary. Sections taken into counting were obtained in every fifth section. **g** Column showed the number of total follicles in the ovary from different treatment groups. **h** Column displayed the number of DDX4-positive primordial follicles in the ovarian section. Data are presented as means ± SEM, *p < 0.05, **p < 0.01, ***p < 0.001, ****p < 0.0001 vs. Bu/Cy group; ^#^*p* < 0.05, ^##^*p* < 0.01, ^####^*p* < 0.0001 vs. SA-BG group; ^$^ *p* < 0.05 vs. SA-BG/hAECs group

To evaluate the effect of the peri-ovary transplantation of SA-BG/hAECs and SA-BG/CM composites on ovarian function, H&E staining was conducted at day 28 following transplantation. Results showed that Bu/Cy significantly damaged the ovarian architecture and caused the loss of follicles at different stages. However, healthy follicles were observed in the different treatment groups, including SA-BG, SA-BG/hAECs, or SA-BG/CM groups (Fig. 2a).

According to the results of follicle count, the number of follicles in Bu/Cy mice was significantly decreased compared with that in the Sham group. When mice were transplanted with SA-BG or SA-BG/hAECs after chemotherapy, the number of primordial follicles significantly increased compared to that in the Bu/Cy control group (*p* < 0.05, Fig. 2c); however, no difference was observed in the SA-BG/CM group. Ovaries in SA-BG/hAECs and SA-BG/CM groups had significantly more primary follicles than those in both Bu/Cy and SA-BG groups (*p* < 0.05, Fig. 2d). In addition, there was also an increasing tendency of the number of secondary follicles in SA-BG/hAECs and SA-BG/CM, although these differences were not statistically significant (Fig. 2e). Moreover, compared to that in the Bu/Cy control group, the number of antral follicles in the SA-BG/CM group was significantly increased (*p* < 0.05, Fig. 2f). The total number of follicles obviously increased in different treatment groups (Fig. 2g). In addition, DDX4 was used to identify the primordial follicle in the cortical area of the ovary [28]. An increased number of primordial follicles (DDX4 positive) was observed in the SA-BG/hAECs group compared with that in the Bu/Cy control group and SA-BG group (*p* < 0.05, Fig. 2h). These results demonstrated that SA-BG or SA-BG/hAECs could protect the primordial follicle pool and SA-BG-loaded hAECs or CM could improve the follicle development in chemotherapy-induced POF mice.

### SA-BG-loaded hAECs/CM protected the function of granulosa cell from chemotherapy-induced damage

**Fig. 3:**
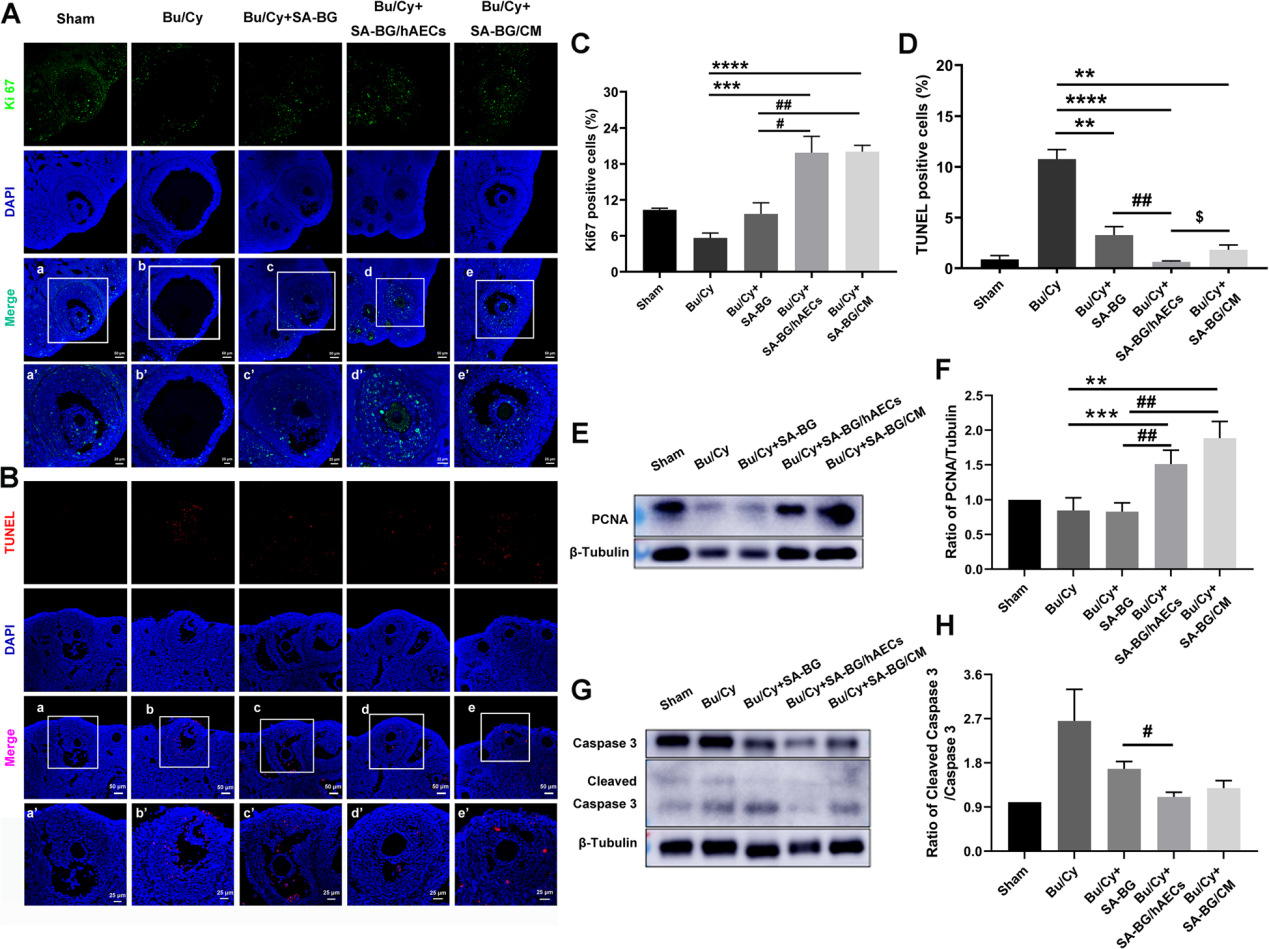
Immunofluorescent staining and detection of related proteins at day 3 after transplantation. **a** Representative images of proliferative cells (Ki67 positive, green color). Scale bars 50 μm and 25 μm. **b** Representative images of apoptotic cells (TUNEL positive, red color) in different groups. Scale bars 50 μm and 25 μm. **c** Column showed the proliferative rate of granulosa cells in the antral follicles. **d** Column displayed the apoptosis rate of granulosa cells in the antral follicles. Nuclei were counterstained with DAPI (blue). **e**, **f** Expression of PCNA protein in different groups. **g**, **h** Expression of apoptotic proteins in different groups (*n* = 3). Data are presented as the mean ± SEM. *p < 0.05, **p < 0.01, ***p < 0.001, ****p < 0.0001 vs. Bu/Cy group; ^##^*p* < 0.01 vs. SA-BG group; ^$^*p* < 0.05 vs. SA-BG/hAECs group

To further determine the role of SA-BG/hAECs or SA-BG/CM in improving the follicle development, the proliferation and apoptosis of cells in ovaries were analyzed with samples obtained from mice at day 3 after transplantation. Results of fluorescence staining showed that transplantation of SA-BG/hAECs and SA-BG/CM significantly increased the percentage of Ki67-positive cells in antral follicles compared to Bu/Cy and SA-BG groups (*p* < 0.05, Fig. 3a, c). In addition, the protein level of PCNA was evaluated by western blotting, and there was a significant increase in the SA-BG/hAECs and SA-BG/CM groups compared to the Bu/Cy and SA-BG groups (*p* < 0.05, Fig. 3e, f). These results demonstrated SA-BG-loaded hAECs/CM could enhance the proliferation of granulosa cells in the injured ovaries. Furthermore, the number of TUNEL-positive cells in SA-BG, SA-BG/hAECs, and SA-BG/CM groups was reduced compared to the Bu/Cy control group (*p* < 0.05, Fig. 3b, d). Accordingly, the protein level ratio of cleaved Caspase3 to Caspase3 showed a decreased tendency in SA-BG, SA-BG/hAECs, and SA-BG/CM groups (Fig. 3g, h). Notably, SA-BG/hAECs led to more prominent inhibition of cell apoptosis than other groups. Our results suggested that the application of SA-BG/hAECs or SA-BG/CM to periovarian tissues promoted the proliferation of granulosa cells in antral follicles and protected them from chemotherapy-induced apoptosis at the early stage of transplantation.

### SA-BG-loaded hAECs/CM enhanced angiogenesis in injured ovaries

**Fig. 4:**
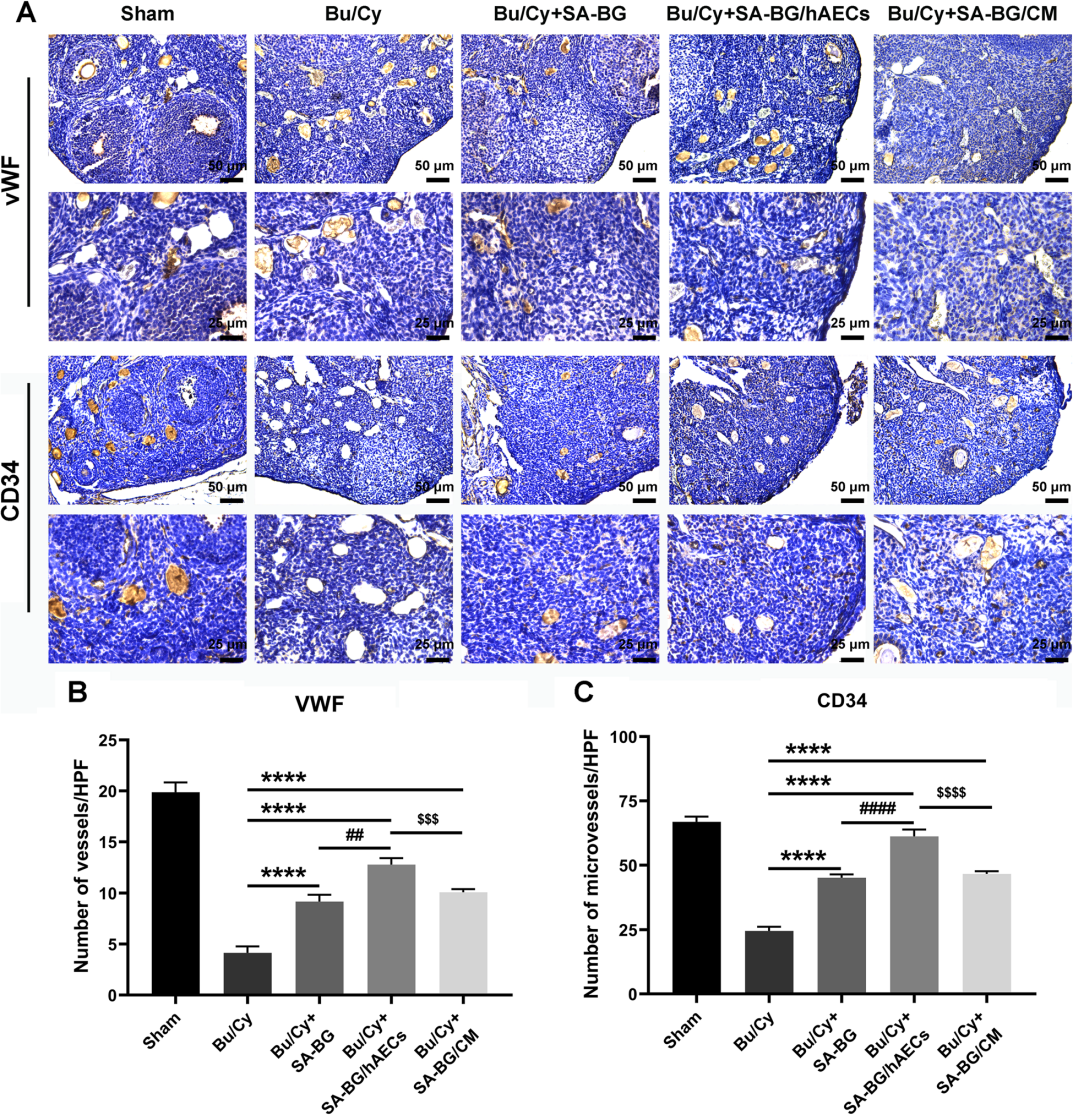
The detection of ovarian angiogenesis in different treatment groups at day 28 after transplantation. **a** Histochemical images displayed vWF- and CD34-positive cells in ovarian stroma from different treatment groups. Scale bar 50 μm and 25 μm. **b** MVD quantification determined by CD34- and vWF-positive cells showed the number of microvessels in ovarian sections of the different groups. *p < 0.05 vs. Bu/Cy group; ^##^*p* < 0.01, ^####^*p* < 0.0001 vs. SA-BG group; ^$$$^*p* < 0.0005, ^$$$$^*p* < 0.0001 vs. SA-BG/hAECs group

Although the study has indicated that granulosa cell apoptosis is the key mechanism responsible for follicle loss, acute vessel damage is confirmed to be involved in ovarian damage caused by chemotherapy [29]. To evaluate the effect of SA-BG/hAECs or SA-BG/CM on angiogenesis in chemotherapy-damaged ovaries, we detected the protein expression of vWF and CD34 in different treatment groups at day 28 after transplantation (Fig. 4a). Moreover, the density of the microvessels (MVD) in the ovarian stroma was evaluated by counting vWF-positive or CD34-positive cells per high-power field. Results showed that the MVD was significantly decreased in the Bu/Cy group compared to the Sham group (*p* < 0.05). However, all three groups including SA-BG, SA-BG/hAECs, and SA-BG/CM showed a significant increase in MVD than that in the Bu/Cy control group (*p* < 0.05). Notably, the SA-BG/hAECs exhibited better effect on angiogenesis than the SA-BG group and SA-BG/CM group (*p* < 0.05, Fig. 4b, c). These results indicated that SA-BG, SA-BG/hAECs, and SA-BG/CM transplantation could promote ovarian angiogenesis in POF mice.

### SA-BG-loaded hAECs/CM promoted the tube formation of hUVECs in vitro

**Fig. 5:**
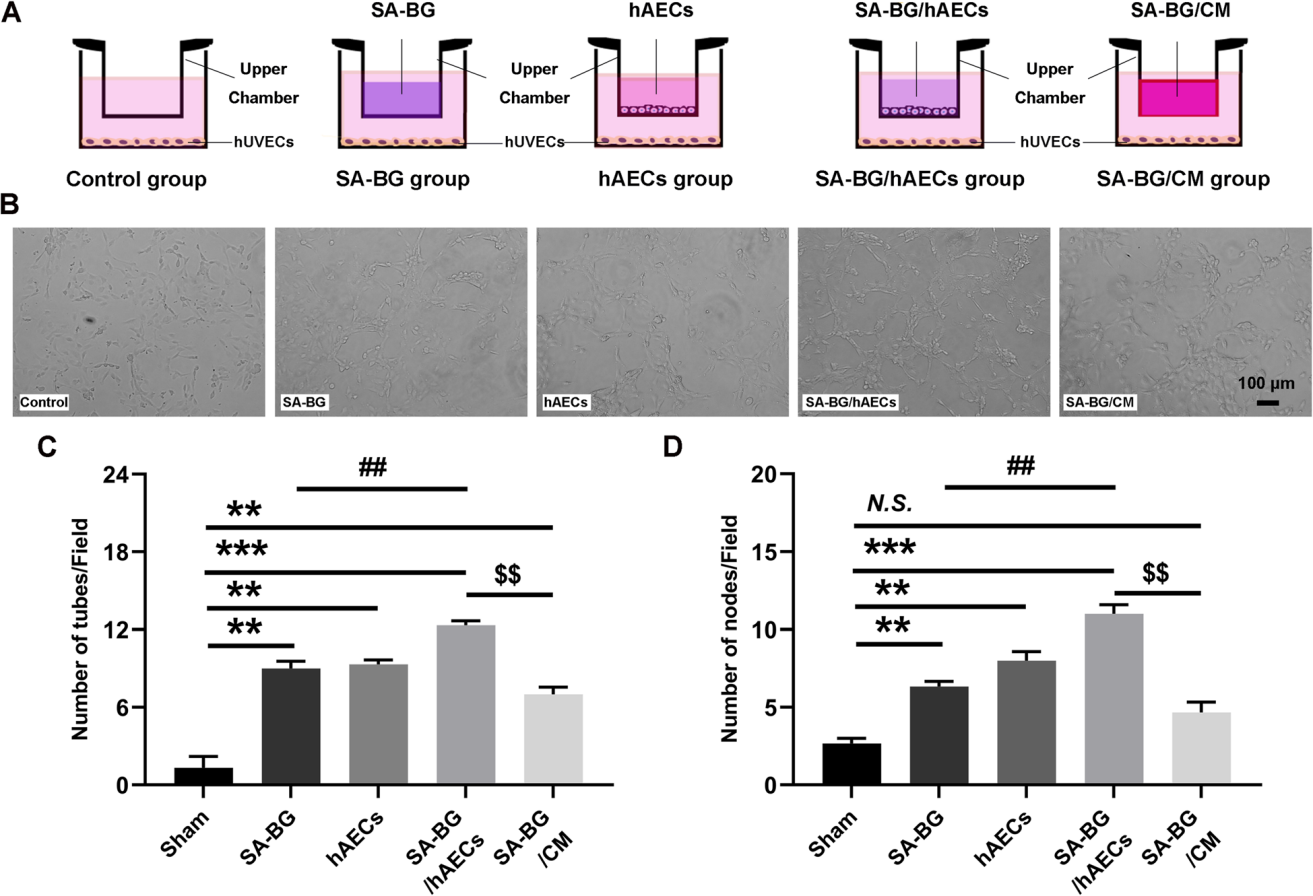
Tube formation assay of hUVECs in vitro. **a** Illustration of the patterns in the tube formation assay and representative images of hUVECs in the different groups, including control, SA-BG, hAECs, SA-BG/hAECs, and SA-BG/CM groups, according to substance type in the upper chamber. Scale bar 100 μm. **b** Representative images of the tube formation assay in different treatment groups. **c**, **d** Quantitative analysis of tube formation was performed by counting nodes and tubular structures. Data presented as means ± SEM from 3 independent experiments. **p < 0.01, ***p < 0.001 vs. Bu/Cy group; ^##^*p* < 0.01 vs. SA-BG group; ^$$^*p* < 0.001 vs. SA-BG/hAECs group

To better clarify the effects of SA-BG, SA-BG/hAECs, and SA-BG/CM on angiogenesis, the tube formation assay of hUVECs was conducted in a coculture system in vitro (Fig. 5a). As shown in Fig. 5b, the increased capillary tube formation of hUVECs was observed in SA-BG, hAECs, SA-BG/hAECs, and SA-BG/CM groups. Statistical results showed that both SA-BG and hAECs could promote tube formation by counting the number of tubes and nodes; moreover, hAECs encapsulated with SA-BG could further enhance this positive effect. Furthermore, compared to SA-BG/CM, SA-BG/hAECs also exhibited better effect on the tube formation (*p* < 0.05, Fig. 5c, d). These results demonstrated that encapsulating hAECs could facilitate the effect of SA-BG on inducing the formation of the capillary tube in a paracrine manner.

### SA-BG extracts stimulated the secretion of pro-angiogenic factors by hAECs

**Fig. 6:**
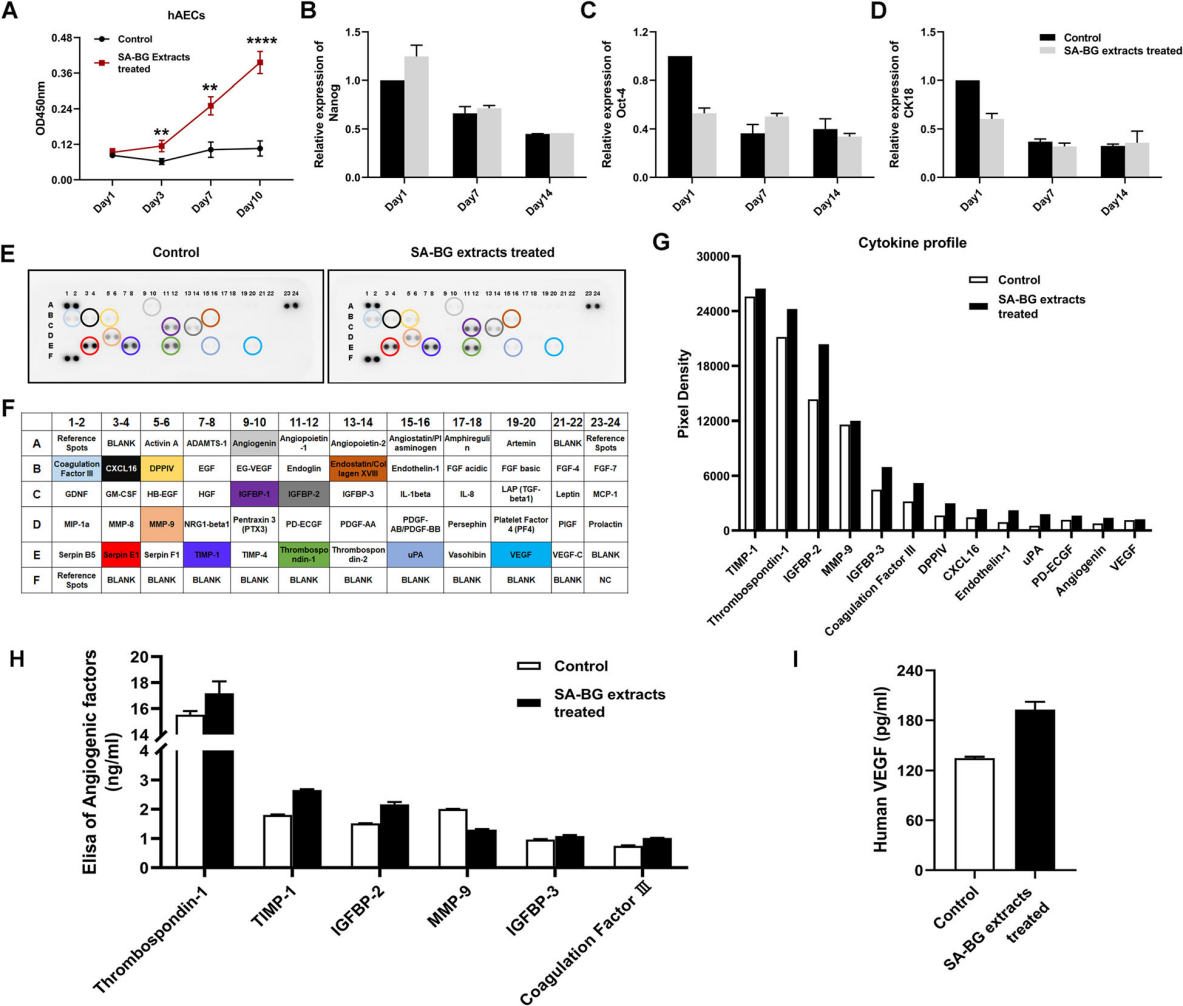
The effect of SA-BG extracts on the biological characterization and paracrine capacity of hAECs. **a** The viability of hAECs cultured with SA-BG extracts was detected by CCK-8 assay. **b**–**d** Expression of stemness (Oct-4 and Nanog) and epithelial (CK18) genes of hAECs cultured with SA-BG extracts at different time points. **e**, **f** The results of cytokine array of CM from hAECs and SA-BG extract-treated hAECs, respectively. **g** Column displayed the higher expression of cytokines in the SA-BG extract-treated hAECs than in the control hAECs. **h**, **i** The results of quantification of angiogenic factors released from SA-BG extract-treated hAECs and control hAECs by ELISA

To analyze whether BG, the bioactive component in SA-BG composite hydrogel, could affect the characteristics of hAECs, SA-BG extracts were harvested and used to culture hAECs. As shown in Fig. 6a, SA-BG extracts significantly enhanced the viability of hAECs compared with the control group (*p* < 0.05). To explore the effect of SA-BG extracts on stemness and epithelial characteristics of hAECs, qRT-PCR assays were performed to analyze the gene expression of Oct-4, Nanog, and CK18. Results showed that SA-BG extracts had no effect on the stemness and epithelial characteristics of hAECs (Fig. 6b–d).

Because that SA-BG-loaded hAECs significantly increased angiogenesis in vivo and vitro, thus, we further explored the effect of SA-BG extracts on the secretion of pro-angiogenesis cytokines from hAECs. A human cytokine array was used to detect the release pattern of angiogenesis-related cytokines in the CM derived from hAECs and SA-BG extract-treated hAECs, respectively. Results showed that a large number of cytokines, including Serpin E1, TIMP-1, Thrombospondin-1, IGFBP-2, MMP-9, IGFBP-3, coagulation factor III, DPPIV, CXCL16, Endothelin-1, angiogenin, and VEGF, were detected in the CM derived from hAECs (Fig. 6e–g). Among these cytokines, the expression levels of TIMP-1, Thrombospondin-1, IGFBP-2, MMP-9, IGFBP-3, coagulation factor III, and VEGF were further determined using ELISA (Fig. 6h, i), which were increased in the CM from SA-BG extract-treated hAECs. These results demonstrated that SA-BG extracts could stimulate hAECs to secrete the high levels of angiogenic factors, which might be beneficial for angiogenesis and vascularization in injured ovarian tissues.

## Discussion

In the field of regenerative medicine, SA has been widely used to encapsulate chemicals and pharmaceuticals due to its biocompatibility, safety, degradability, and in vivo stability [30]. BG is a bioactive engineering substance that can be mixed with SA to fabricate SA-BG composite hydrogel beads for use as stem cell carriers that gradually release bioactive ions and stem cells for bone regeneration [31]. SA-BG not only promoted the migration of fibroblasts and endothelial cells, but also enhanced the vessel formation of endothelial cells, which was beneficial for angiogenesis and vascularization in chronic wound healing [32, 33]. hAECs, as alternative stem cell, have attracted great attention and exhibit extremely high therapeutic potential for treating many diseases [8]. In previous studies, we have demonstrated that hAECs and hAEC derivatives (CM and exosomes) play important roles in restoring ovarian function and fertility [9, 12, 13]. However, it is still unknown whether SA-BG-loaded hAECs/CM could be used to repair ovarian function in chemotherapy-induced POF mice. In this study, we first established an original approach of SA-BG encapsulating hAECs or CM transplantation around damaged ovaries and investigated whether hAECs or CM encapsulated in SA-BG composite hydrogel could play effective roles in protecting ovarian function from chemotherapy-induced damage.

It is well known that the viability of grafted stem cells in injured tissues extensively affects the repair effect. A study has demonstrated that following transplantation, stem cells usually die due to the ischemic and inflammatory microenvironments in the injured tissues [34]. In addition, the proliferative ability of hAECs was limited due to the lack of telomerase expression [35], which substantially inhibited the therapeutic effect of stem cells [34, 36, 37]. In the current study, we found that hAECs could survive in SA-BG composite hydrogel, indicating that SA-BG is not toxic to hAECs. Meanwhile, we observed that hAECs encapsulated in SA-BG composite hydrogel exhibited a dot-like appearance, which was consistent with a previous study of BMSCs encapsulated in SA-BG [31, 33]. However, our current results found that SA-BG extracts had no obvious effect on the epithelial and stemness characteristics of hAECs.

In the chemotherapy-induced POF mouse model, we transplanted SA-BG, SA-BG/hAECs, and SA-BG/CM around the damaged ovaries and investigated their effects on ovarian function. Previous studies have shown that decreased ovarian function was a consequence of the chemotherapy-induced excessive dormant primordial follicle activation [38], massive granulosa cell apoptosis [39], and persistent vascular damage [29]. Results of follicle count showed that SA-BG/hAECs or SA/BG-CM protected ovarian follicles, especially for primordial follicles, from exhaustion. Compared to SA-BG*/*CM, SA-BG/hAECs produce better efficacy in protecting the primordial follicles, which is beneficial for long-term ovarian function*.* In the early stage of transplantation, the proliferation of granulosa cells significantly increased in the SA-BG/hAECs group; however, chemotherapy-induced granulosa cell apoptosis was inhibited, which contributed to maintaining follicle development.

Acute vessel damage is an important problem in chemotherapy-induced POF that cannot be ignored. Our previous studies have confirmed that hAECs have the potential to protect ovarian vessels against destruction by the paracrine pathway [11, 12]. A study has shown that bioactive ions released from BG in the process of degradation could promote neovascularization, facilitating tissue repair [40]. Herein, angiogenesis in the injured ovaries was further analyzed and results suggested that SA-BG played a synergistic role with hAECs or CM in promoting angiogenesis in vivo. In the tube formation of hUVEC assay, we observed that the numbers of tubes and nodes in hUVECs were significantly increased when cocultured with SA-BG/hAECs or SA-BG/CM than that with hAECs or SA-BG. To elucidate the underlying mechanism, we further conducted CCK-8 and detected the secretion of angiogenic/vasculogenic factors in the CM from hAECs cultured with SA-BG extracts via cytokine array. Results showed that SA-BG extracts not only stimulated the viability of hAECs, but also induced hAECs to generate a significant amount of angiogenesis-associated cytokines. Previous studies have demonstrated that Serpin E1, TIMP-1, insulin-like growth factor binding proteins-2/3 (IGFBP-2/3), and VEGF are important angiogenic factors to facilitate angiogenesis [41–43]. As a consequence of stimulation by these factors, new blood vessels form and start transporting oxygen and nutrients to damaged tissues, which contributes to functional recovery [44]. However, the impact of hAECs on angiogenesis may be influenced by the presence of inflammation lesions [45]. Thus, how the paracrine capacity of hAECs in SA-BG is affected after transplantation into the damaged ovary requires further substantial exploration. Taken together, the current results indicated that SA-BG/hAECs played a synergetic role in promoting angiogenesis and vascularization in the injured ovaries.

hAEC-CM is rich in soluble bioactive cytokines, which are increasingly favored as promising cell-free components in biomaterial development; however, the stability of these bioactive factors over time is not fully clear [46, 47]. In the current study, the protective effect of SA-BG/CM was not superior, which might be related to the insufficient amount and/or the uncertain half-life of the effective components. In a further study, the preparation and collection of CM need to be optimized.

The present study reported that the transplantation of hAECs encapsulated with SA-BG composite hydrogel onto damaged ovaries was an effective technique, and this method did not disrupt the ovarian architecture compared with injection in situ. However, there are several limitations that need to be acknowledged. First, the adhesive properties, proliferative capabilities, and migration and homing abilities of hAECs encapsulated with SA-BG in ovaries could be explored. In addition, to better determine the duration of BG action, the pattern of bioactive ions releasing from the composite hydrogel and the paracrine activity of hAECs in vivo need to be further studied.

## Conclusion

This study integrated the advantages of SA-BG and hAECs to create a locally injected, excellent bioattached, nontoxic and efficient approach of stem cell transplantation for treating chemotherapy-induced ovarian damage. The use of SA/BG-encapsulated hAECs could play a positive role in ovarian function recovery by releasing soluble factors instead of homing to specific organs or establishing cell-cell contacts. Additionally, further study revealed that BG effectively stimulated the paracrine function of hAECs, especially the release of angiogenic factors, which contributed to promoting angiogenesis and follicle development in the damaged ovaries. Our research indicates that the application of hAECs encapsulated in SA-BG composite hydrogel could be a promising alternative for practical transplantation techniques for treating POF.

## Supplementary Information

**Additional file 1: Supplementary Table 1.** PCR primers used to detect gene expression in hAECs.

## Data Availability

Not applicable.

## Abbreviations

hAECs: Human amniotic epithelial cells
POF: Premature ovarian failure
SA-BG: Sodium alginate-bioglass
CM: Conditioned medium
hUVECs: Human umbilical vein epithelial cells
HRT: Hormone replacement therapy
UC-MSCs: Umbilical cord-derived mesenchymal stem cells
BMSCs: Bone marrow stem cells
DMEM: Dulbecco’s modified Eagle’s medium
CK18: Cytokeratin 18
Bu: Busulfan
Cy: Cyclophosphamide
H&E: Hematoxylin and eosin
IHC: Immunohistochemical
HPFs: High-power fields
MVD: Microvessel density
Calcein-AM: Calcein acetoxymethyl ester
PI: Propidium iodide
CCK-8: Cell Counting Kit-8
qRT-PCR: Quantitative real-time polymerase chain reaction
IGFBP-2/3: Insulin-like growth factor binding proteins-2/3
PAI1: Plasminogen activator inhibitor 1
MMPs: Matrix metalloproteinases
ISO: Isotypes
