# Supplementary material for: Sodium alginate-bioglass-encapsulated hAECs restore ovarian function in premature ovarian failure by stimulating angiogenic factor secretion
Source: Stem Cell Res Ther. 2021 Apr 1;12:223. doi: 10.1186/s13287-021-02280-2 (PMC8015041; doi:10.1186/s13287-021-02280-2)
Supplement: Supplementary file 1 — Additional file 1: Supplementary Table 1. PCR primers used to detect gene expression in hAECs. [file 13287_2021_2280_MOESM1_ESM.docx]

Table 1. PCR primers used to detect gene expression in hAECs.

| **Gene symbol** | **Primer sequence** | | | **Fragment**  **size (bp)** |
| --- | --- | --- | --- | --- |
| Nanog | Forward: | TTTGTGGGCCTGAAGAAAACT |  | 116 |
|  | Reverse: | AGGGCTGTCCTGAATAAGCAG |  |  |
| OCT-4 | Forward: | GGGAGATTGATAACTGGTGTGTT |  | 144 |
|  | Reverse: | GTGTATATCCCAGGGTGATCCTC |  |  |
| CK18 | Forward: | GTTGACCGTGGAGGTAGATGC |  | 86 |
|  | Reverse: | GAGCCAGCTCGTCATATTGGG |  |  |
| ACTIN | Forward: | CATGTACGTTGCTATCCAGGC |  | 250 |
|  | Reverse: | CTCCTTAATGTCACGCACGAT |  |  |
